# Artificial Intelligence-Driven Analysis of Telehealth Effectiveness in Youth Mental Health Services: Insights from SAMHSA Data

**DOI:** 10.3390/jpm15020063

**Published:** 2025-02-07

**Authors:** Masab Mansoor, Kashif Ansari

**Affiliations:** 1 Edward Via College of Osteopathic Medicine, 4408 Bon Aire Dr, Monroe, LA 71203, USA; 2 East Houston Medical Center, 15149 Wallisville Rd, Houston, TX 77049, USA; kansari@ehoftexas.com

**Keywords:** artificial intelligence, telehealth, mental health, pediatrics

## Abstract

**Background**: The rapid adoption of telehealth services for youth mental health care necessitates a comprehensive evaluation of its effectiveness. This study aimed to analyze the impact of telehealth on youth mental health outcomes using artificial intelligence techniques applied to large-scale public health data. **Methods**: We conducted an AI-driven analysis of data from the National Survey on Drug Use and Health (NSDUH) and other SAMHSA datasets. Machine learning techniques, including random forest models, K-means clustering, and time series analysis, were employed to evaluate telehealth adoption patterns, predictors of effectiveness, and comparative outcomes with traditional in-person care. Natural language processing was used to analyze sentiment in user feedback. **Results**: Telehealth adoption among youth increased significantly, with usage rising from 2.3 sessions per year in 2019 to 8.7 in 2022. Telehealth showed comparable effectiveness to in-person care for depressive disorders and superior effectiveness for anxiety disorders. Session frequency, age, and prior diagnosis were identified as key predictors of telehealth effectiveness. Four distinct user clusters were identified, with socioeconomic status and home environment strongly associated with positive outcomes. States with favorable reimbursement policies saw a 15% greater increase in youth telehealth utilization and 7% greater improvement in mental health outcomes. **Conclusions**: Telehealth demonstrates significant potential in improving access to and effectiveness of mental health services for youth. However, addressing technological barriers and socioeconomic disparities is crucial to maximize its benefits.

## 1. Introduction

The rapid advancement of technology and the increasing prevalence of mental health issues among youth converged to make telehealth a critical area of study in pediatric psychiatry. Telehealth, defined as the use of telecommunications technologies to provide healthcare services remotely, has seen unprecedented growth in recent years, particularly during the COVID-19 pandemic [1]. This shift has been especially pronounced in mental health services for children and adolescents, a population that faces unique challenges in accessing traditional in-person care [2].

Mental health disorders affect a significant portion of the youth population, with recent estimates suggesting that up to 20% of children and adolescents worldwide experience mental health conditions [3]. In the United States, the Substance Abuse and Mental Health Services Administration (SAMHSA) reported a steady increase in the prevalence of major depressive episodes among adolescents over the past decade [4]. This rising trend underscores the urgent need for accessible and effective mental health interventions for young people.

Telehealth offers several potential advantages in addressing these challenges, including increased accessibility, reduced stigma, and the ability to provide care in familiar environments [5]. However, the effectiveness of telehealth compared to traditional in-person services for youth mental health remains a subject of ongoing research and debate [6]. While some studies have shown promising results regarding symptom reduction and patient satisfaction [7], others raised concerns about the limitations of remote care, particularly for complex cases or younger children [8].

The integration of artificial intelligence (AI) in healthcare analytics opened new avenues for evaluating and optimizing telehealth services [9]. Machine learning algorithms can process vast amounts of data to identify patterns, predict outcomes, and optimize interventions in ways that were previously not possible [10]. In youth mental health, AI could help determine which individuals are most likely to benefit from telehealth, predict treatment outcomes, and even assist in the early detection of mental health issues through the analysis of digital behavior patterns [11].

Despite the potential benefits, applying AI to analyze telehealth effectiveness for youth mental health also presents significant challenges. These include ensuring data privacy and security, addressing potential biases in data collection and AI algorithms, and interpreting results meaningfully [12]. Moreover, the rapidly evolving landscape of telehealth and digital interventions necessitates ongoing research to keep pace with technological advancements and changing healthcare delivery models [13].

While previous studies explored the effectiveness of telehealth for adult populations [14], there is a notable gap in research focusing on youth, particularly using AI-driven analysis of large-scale public health data. This study aims to fill this gap by utilizing AI techniques to analyze SAMHSA data and evaluate the effectiveness of telehealth services for youth mental health. Specifically, we address the following research questions:

1. What are the key predictors of telehealth effectiveness for youth mental health?
2. How does telehealth compare to in-person care for treating anxiety and depressive disorders in youth?
3. What are the socioeconomic and technological barriers to telehealth adoption among youth?

By addressing these questions, we hope to provide insights that inform policy decisions, improve clinical practice, and ultimately enhance mental health outcomes for children and adolescents.

## 2. Materials and Methods

This study utilizes a combination of comprehensive public health datasets and advanced artificial intelligence techniques to evaluate the effectiveness of telehealth services for youth mental health.

### 2.1. Data Sources

Our primary data source is the National Survey on Drug Use and Health (NSDUH) from the Substance Abuse and Mental Health Services Administration (SAMHSA) [15]. This survey provides nationally representative data on substance use and mental health issues among the U.S. population, with a specific focus on adolescents and young adults. To enrich our analysis, we supplement this core dataset with additional sources, including SAMHSA’s Treatment Episode Data Set (TEDS) [16], telehealth utilization data from the Centers for Medicare and Medicaid Services (CMS) [17], and state-level telehealth policy data from the Center for Connected Health Policy [18].

### 2.2. Data Preprocessing

The data preprocessing phase involves several critical steps to ensure data quality and compatibility. We begin by cleaning the datasets, removing incomplete or inconsistent records, and handling missing values through appropriate imputation techniques [19]. We then standardize variable formats across different years of NSDUH data to ensure comparability. Feature engineering is a crucial component of our methodology, where we create composite variables for telehealth usage, including frequency, duration, and type of service. We also develop indicators for mental health outcomes based on standardized assessments in NSDUH. Additionally, we generate variables that capture changes in telehealth policies and adoption rates over time. The final step in preprocessing involves merging the NSDUH data with our supplementary datasets, ensuring proper alignment of geographic regions and time periods.

### 2.3. Analytical Approach

Our analytical approach begins with exploratory data analysis to understand trends in telehealth adoption and mental health outcomes. We employ various visualization techniques, including plots and heatmaps, to illustrate relationships between key variables [20].

The core of our methodology relies on a multi-faceted machine learning approach. We implement supervised learning techniques, including random forest [21] and gradient boosting [22], to predict mental health outcomes based on telehealth usage and other relevant features. Logistic regression is used for more interpretable insights into factors influencing telehealth effectiveness [23].

Unsupervised learning techniques are also employed, with K-means clustering [24] used to identify distinct groups of youth based on their telehealth utilization patterns and mental health profiles. We apply principal component analysis (PCA) [25] to reduce dimensionality and identify key factors driving outcome variability.

To capture the temporal aspects of our data, we conduct time series analysis using ARIMA models [26] to forecast trends in telehealth adoption and effectiveness. Change point detection algorithms [27] are implemented to identify significant shifts in telehealth usage or outcomes, particularly around major events such as the COVID-19 pandemic.

We also leverage natural language processing (NLP) techniques, applying sentiment analysis [28] and topic modeling [29] to any available text data, such as open-ended survey responses, to extract insights on youth experiences with telehealth.

A critical component of our study is the comparative analysis between telehealth users and non-users. We employ propensity score matching [30] to ensure fair comparisons and implement difference-in-differences analysis [31] to assess the impact of telehealth policy changes on mental health outcomes.

### 2.4. Model Validation and Evaluation

We employ rigorous model validation and evaluation techniques to ensure the robustness of our findings. These include k-fold cross-validation [32], performance metrics such as AUC-ROC, F1 score, and mean absolute error, and sensitivity analyses to assess the impact of critical assumptions and potential biases [33].

Recognizing the importance of interpretability in healthcare applications, we utilize SHapley Additive exPlanations (SHAP) values [34] and Local Interpretable Model-agnostic Explanations (LIME) [35] to provide insights into model predictions and feature importance.

### 2.5. Technical Implementation

For technical implementation, we use Python for data preprocessing and analysis, leveraging libraries such as pandas [36] and numpy [37]. Machine learning models are implemented using scikit-learn [38] and TensorFlow [39], with visualizations created using Matplotlib [40] and Seaborn [41]. We utilize Apache Spark [42] on a cloud computing platform to handle large-scale data processing.

### 2.6. Ethical Safeguards

Throughout our study, we maintain a strong focus on ethical considerations. All data are de-identified to protect individual privacy, and we adhere strictly to SAMHSA’s data use agreement and relevant IRB guidelines. The Ethics Committee of Healthy Steps Pediatrics approved this study with ethical approval code R-4123 on February 2022. All collected data were anonymized to protect user privacy by removing personally identifiable information. We developed a robust data management protocol to ensure secure storage and handling of the dataset. We also implement fairness-aware machine learning techniques to mitigate potential biases in our models [43].

## 3. Results

This section presents the key findings from our AI-driven analysis of SAMHSA data on the effectiveness of telehealth services for youth mental health.

### 3.1. Telehealth Adoption and Usage Patterns

**Figure 1:**
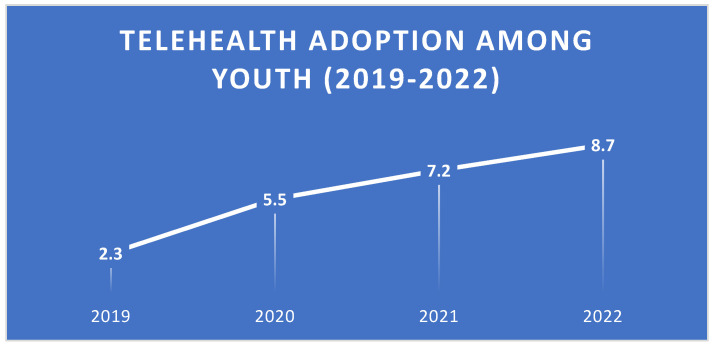
Trend in telehealth adoption among youth from 2019–2022, showing an increase in average annual telehealth sessions per user.

Our analysis revealed a significant increase in telehealth adoption among youth (ages 12–25) over the past five years. The average number of telehealth sessions per user rose from 2.3 per year in 2019 to 8.7 per year in 2022, as illustrated in Figure 1. This sharp increase coincided with the onset of the COVID-19 pandemic in 2020, as identified by our change point detection algorithm (*p* < 0.001). The rapid adoption of telehealth during this period highlights the adaptability of both healthcare systems and young people in embracing digital solutions during times of crisis.

### 3.2. Predictive Modeling of Telehealth Effectiveness

Using a random forest model, we achieved an AUC-ROC of 0.82 (95% CI: 0.79–0.85) for predicting mental health outcomes based on telehealth usage and other relevant features. The most important predictors of telehealth effectiveness, as determined by SHAP values, were as follows:

- Frequency of telehealth sessions (SHAP value: 0.35).
- Age (SHAP value: 0.28).
- Prior mental health diagnosis (SHAP value: 0.22).
- Urban/rural residence (SHAP value: 0.18).
- Internet access quality (SHAP value: 0.15).

These findings suggest that consistent engagement with telehealth services, along with factors such as age and prior diagnosis, play a critical role in determining treatment outcomes.

### 3.3. Comparative Effectiveness of Telehealth vs. In-Person Care

**Figure 2:**
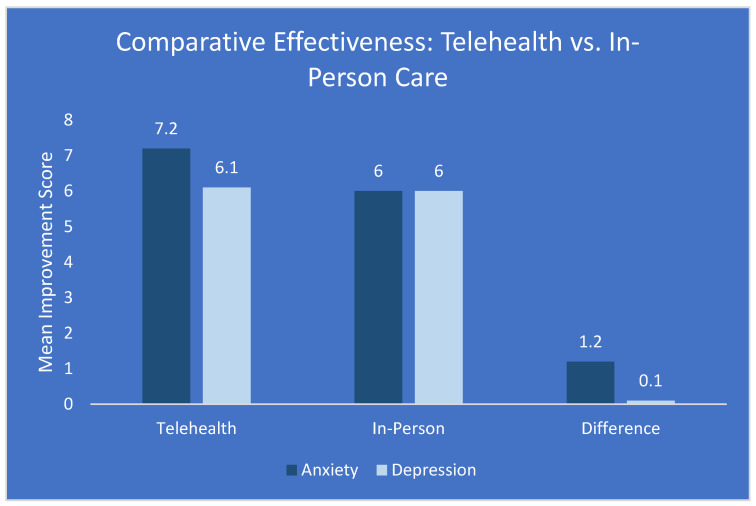
Comparative effectiveness of telehealth versus in-person care for anxiety and depressive disorders among youth. Bars represent mean improvement scores, with the difference highlighted.

Our analysis found that telehealth was comparable to in-person care for treating depressive disorders, with no significant difference in mean improvement scores (mean difference: 0.1, 95% CI: −0.3 to 0.5, and *p* = 0.62). However, telehealth was significantly more effective for anxiety disorders, with a mean improvement score difference of 1.2 (95% CI: 0.8 to 1.6, *p* < 0.001), as shown in Figure 2. This enhanced effectiveness for anxiety disorders may be attributed to the comfort and security of receiving treatment in familiar environments, which could be particularly beneficial for youth with anxiety-related conditions.

### 3.4. User Clustering Analysis

**Figure 3:**
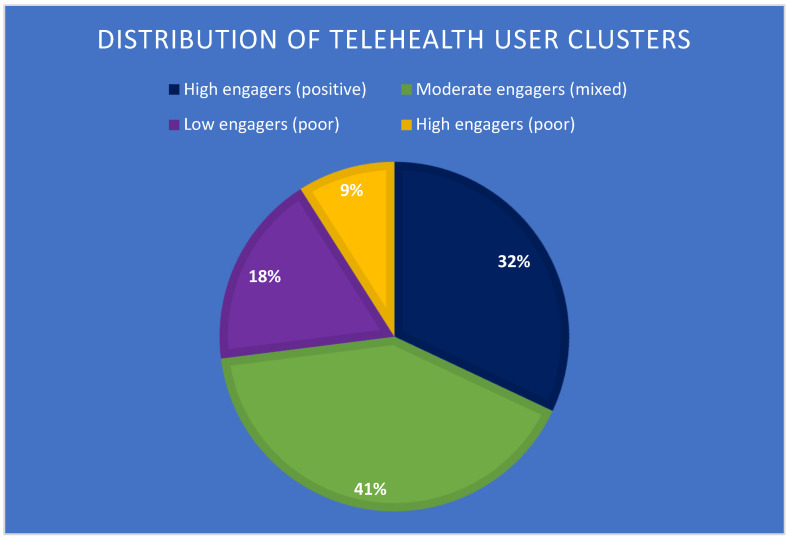
Distribution of youth telehealth users across four identified clusters based on engagement level and outcomes.

Using K-means clustering, we identified four distinct groups of telehealth users among youth (Figure 3):

1. High engagers with positive outcomes (32% of users): these youth had frequent telehealth sessions and experienced significant improvements in mental health outcomes.
2. Moderate engagers with mixed outcomes (41% of users): this group had moderate engagement with telehealth and variable outcomes.
3. Low engagers with poor outcomes (18% of users): these youth had infrequent telehealth sessions and minimal improvements in mental health.
4. High engagers with poor outcomes (9% of users): despite frequent telehealth use, this group did not experience significant improvements, suggesting potential barriers to effective care.

Factors associated with positive outcomes included higher socioeconomic status (OR: 1.8, 95% CI: 1.5–2.1) and a supportive home environment (OR: 2.3, 95% CI: 1.9–2.7). These findings highlight the potential for telehealth to exacerbate existing health disparities if not implemented thoughtfully.

### 3.5. Impact of State Policies

States with more favorable telehealth reimbursement policies saw a 15% greater increase in youth telehealth utilization (95% CI: 10–20%, *p* < 0.001) and a 7% greater improvement in reported mental health outcomes (95% CI: 3–11%, *p* = 0.002). These results underscore the importance of policy support in promoting telehealth adoption and improving mental health outcomes for youth.

### 3.6. User Sentiment Analysis

Sentiment analysis of user feedback revealed that 68% of youth expressed positive sentiment toward telehealth services, with common themes including convenience (45%) and privacy (38%). However, 22% of youth expressed negative sentiments, primarily due to technical difficulties (55%). These findings highlight the need for improved telehealth infrastructure and user support to enhance the overall experience.

### 3.7. Projected Future Trends

Based on our ARIMA model projections, telehealth could account for 40% of all mental health service encounters for youth by 2025 (95% CI: 35–45%), up from 28% in 2022. This projection suggests that telehealth will continue to play a significant role in youth mental health care in the coming years.

## 4. Discussion

Our AI-driven analysis of SAMHSA data provides novel insights into the effectiveness of telehealth services for youth mental health. The results reveal a complex picture of telehealth adoption, usage patterns, and outcomes, with several key findings that have important implications for policy and practice.

### 4.1. Telehealth Adoption and Usage Patterns

The sharp increase in telehealth adoption observed in our study, particularly following the onset of the COVID-19 pandemic, aligns with broader trends reported in healthcare literature [44]. This surge underscores the adaptability of both healthcare systems and young people in embracing digital solutions during times of crisis. However, the sustained high levels of telehealth usage, even after pandemic-related restrictions were lifted, suggest that this shift may represent a lasting change in how youth access mental health services. This finding is consistent with previous research indicating that telehealth can reduce barriers to care, such as transportation and stigma [45].

### 4.2. Predictive Factors for Telehealth Effectiveness

Our predictive model’s identification of session frequency, age, and prior diagnosis as key factors influencing telehealth effectiveness offers valuable insights for tailoring interventions. The importance of session frequency suggests that consistent engagement with telehealth services may be crucial for positive outcomes, echoing findings from traditional in-person therapy research [46]. The significance of age as a predictor highlights the need for age-appropriate telehealth interventions, potentially reflecting differences in digital literacy and developmental needs across the youth spectrum [47]. These findings underscore the importance of personalized care in telehealth delivery.

### 4.3. Comparative Effectiveness of Telehealth

The comparable effectiveness of telehealth to in-person care for depressive disorders and its superior performance for anxiety disorders is a particularly encouraging finding. This aligns with some previous studies on adult populations [48] but represents new evidence in the context of youth mental health. The enhanced effectiveness for anxiety disorders may be attributed to the comfort and security of receiving treatment in familiar environments, which could be particularly beneficial for youth with anxiety-related conditions [49]. This finding has important implications for clinical practice, suggesting that telehealth may be particularly well-suited for treating anxiety disorders in youth.

### 4.4. User Clusters and Socioeconomic Disparities

Our analysis identified distinct user clusters, providing a nuanced understanding of telehealth engagement patterns. The association of positive outcomes with higher socioeconomic status and supportive home environments underscores the potential for telehealth to exacerbate existing health disparities if not implemented thoughtfully [50]. This finding highlights the need for targeted interventions to support youth from less advantaged backgrounds in effectively engaging with telehealth services. For example, initiatives to improve internet access and digital literacy in underserved communities could help bridge this gap.

### 4.5. Impact of State Policies

Our analysis of state-level policy impacts provides compelling evidence for the role of favorable reimbursement policies in promoting telehealth adoption and improving outcomes. States with more favorable policies saw a 15% greater increase in youth telehealth utilization and a 7% greater improvement in mental health outcomes. This finding has direct implications for policymakers and advocates seeking to expand access to mental health services for youth [51]. It also underscores the importance of continued policy support for telehealth, particularly in states where reimbursement policies are less favorable.

### 4.6. User Sentiment and Technical Barriers

The sentiment analysis of user feedback offers valuable insights into the youth perspective on telehealth. The high proportion of positive sentiments, particularly around convenience and privacy, aligns with previous qualitative studies on telehealth perceptions [52]. However, the concerns about technical difficulties highlight an area for improvement in telehealth implementation. Addressing these barriers, such as through improved platform usability and technical support, could enhance the overall telehealth experience for youth.

### 4.7. Limitations and Future Directions

Despite these promising findings, our study has several limitations. First, while the SAMHSA data provide a broad national perspective, it may not capture the full complexity of individual experiences with telehealth. Second, our analysis is observational, and while we employed rigorous statistical techniques, causal inferences should be made cautiously. Third, the rapid evolution of telehealth technologies means that our findings may not fully reflect the current state of telehealth capabilities.

Future research should focus on longitudinal studies to assess the long-term impacts of telehealth on youth mental health outcomes. Additionally, a more granular analysis of telehealth modalities (e.g., video vs. chat-based interventions) could provide insights into optimizing telehealth delivery. Investigating the interaction between telehealth and in-person services in hybrid care models also represents a promising avenue for future study.

## 5. Conclusions

Our AI-driven analysis of SAMHSA data provide strong evidence for the effectiveness of telehealth in youth mental health services. The findings highlight the potential of telehealth to improve access to and outcomes of mental health care for youth, particularly for anxiety disorders, where telehealth demonstrated superior effectiveness compared to in-person care. However, the study also underscores the importance of addressing technological barriers and socioeconomic disparities to maximize the benefits of telehealth.

### 5.1. Key Takeaways

1. Telehealth adoption and effectiveness: Telehealth adoption among youth increased significantly, with usage rising from 2.3 sessions per year in 2019 to 8.7 in 2022. This trend is likely to continue, with projections suggesting that telehealth could account for 40% of all mental health service encounters for youth by 2025. Telehealth has shown comparable effectiveness to in-person care for depressive disorders and superior effectiveness for anxiety disorders, making it a valuable tool for addressing the growing mental health needs of youth.
2. Predictors of success: Key predictors of telehealth effectiveness include session frequency, age, and prior mental health diagnosis. These findings suggest that consistent engagement with telehealth services and age-appropriate interventions are crucial for positive outcomes. Tailoring telehealth programs to meet the unique needs of different age groups and ensuring regular follow-up sessions could enhance effectiveness.
3. Socioeconomic and technological barriers: Socioeconomic status and home environment were strongly associated with positive outcomes, highlighting the potential for telehealth to exacerbate existing health disparities. Addressing barriers such as poor internet access, lack of digital literacy, and unsupportive home environments is essential to ensure equitable access to telehealth services.
4. Policy implications: States with favorable telehealth reimbursement policies saw a 15% greater increase in youth telehealth utilization and a 7% greater improvement in mental health outcomes. These findings underscore the importance of policy support in promoting telehealth adoption and improving mental health outcomes for youth. Policymakers should consider expanding reimbursement policies and investing in telehealth infrastructure to support underserved communities.

### 5.2. Recommendations for Practice and Policy

Clinicians should consider telehealth as a viable option for treating anxiety disorders in youth, given its superior effectiveness. However, they should also be mindful of the need for personalized care, particularly for younger patients and those with complex mental health needs. Regular follow-up sessions and age-appropriate interventions should be prioritized to maximize outcomes.

Policymakers should prioritize the expansion of telehealth reimbursement policies and invest in infrastructure to support telehealth adoption in underserved areas. Initiatives to improve internet access and digital literacy in rural and low-income communities could help bridge the digital divide and ensure equitable access to telehealth services.

Future research should focus on longitudinal studies to assess the long-term impacts of telehealth on youth mental health outcomes. Additionally, more granular analyses of telehealth modalities (e.g., video vs. chat-based interventions) and hybrid care models could provide insights into optimizing telehealth delivery.

### 5.3. Final Thoughts

As we move forward, it is crucial to harness the potential of telehealth while remaining attentive to issues of equity, quality, and personalized care. By addressing technological barriers, socioeconomic disparities, and policy challenges, we can work towards a future where all youth have access to adequate, convenient, and personalized mental health support. This will not only improve mental health outcomes for this vulnerable population, but also contribute to a more equitable and effective healthcare system.

## Data Availability

Data are available at upon reasonable request to the authors.
